# Comparing the Effects of Gamification and Teach-Back Training Methods on Adherence to a Therapeutic Regimen in Patients After Coronary Artery Bypass Graft Surgery: Randomized Clinical Trial

**DOI:** 10.2196/22557

**Published:** 2021-12-10

**Authors:** Banafsheh Ghorbani, Alun C Jackson, Mohammad Noorchenarboo, Mohammad H Mandegar, Farshad Sharifi, Zohrehsadat Mirmoghtadaie, Fatemeh Bahramnezhad

**Affiliations:** 1 School of Nursing and Midwifery Tehran University of Medical Sciences Tehran Iran; 2 Australian Centre for Heart Health Melbourne Australia; 3 Faculty of Health Deakin University Geelong Australia; 4 Centre on Behavioural Health Hong Kong University Hong Kong China; 5 Tehran University of Medical Sciences Tehran Iran; 6 Department of Cardiac Surgery Shariati Hospital Tehran University of Medical Sciences Tehran Iran; 7 Elderly Health Research Center Endocrinology and Metabolism Population Sciences Research Institute Tehran University of Medical Sciences Tehran Iran; 8 Shaheed Beheshti University of Medical Sciences Tehran Iran; 9 School of Nursing & Midwifery, Nursing and Midwifery Care Research Center Spiritual Health Group, Research Center of Quran, Hadith and Medicine Tehran University of Medical Sciences Tehran Iran

**Keywords:** teach back, gamification, treatment regimen, coronary artery bypass graft, patient training

## Abstract

**Background:**

Patients undergoing coronary artery bypass graft surgery (CABGS) may fail to adhere to their treatment regimen for many reasons. Among these, one of the most important reasons for nonadherence is the inadequate training of such patients or training using inappropriate methods.

**Objective:**

This study aimed to compare the effect of gamification and teach-back training methods on adherence to a therapeutic regimen in patients after CABGS.

**Methods:**

This randomized clinical trial was conducted on 123 patients undergoing CABGS in Tehran, Iran, in 2019. Training was provided to the teach-back group individually. In the gamification group, an app developed for the purpose was installed on each patient’s smartphone, with training given via this device. The control group received usual care, or routine training. Adherence to the therapeutic regimen was assessed using a questionnaire on adherence to a therapeutic regimen (physical activity and dietary regimen) and an adherence scale as a pretest and a 1-month posttest.

**Results:**

One-way analysis of variance (ANOVA) for comparing the mean scores of teach-back and gamification training methods showed that the mean normalized scores for the dietary regimen (*P*<.001, *F*=71.80), movement regimen (*P*<.001, *F*=124.53), and medication regimen (*P*<.001, *F*=9.66) before and after intervention were significantly different between the teach-back, gamification, and control groups. In addition, the results of the Dunnett test showed that the teach-back and gamification groups were significantly different from the control group in all three treatment regimen methods. There was no statistically significant difference in adherence to the therapeutic regimen between the teach-back and control groups.

**Conclusions:**

Based on the results of this study, the use of teach-back and gamification training approaches may be suggested for patients after CABGS to facilitate adherence to the therapeutic regimen.

**Trial Registration:**

Iranian Registry of Clinical Trials IRCT20111203008286N8; https://en.irct.ir/trial/41507

## Introduction

### Background

The most important objective of coronary artery bypass graft surgery (CABGS) is to improve the patient’s quality of life by reducing angina symptoms and maintaining coronary circulation [1,2]. However, this approach also has complications, despite its many benefits. For example, patients may be exposed to side effects, such as respiratory problems, atelectasis, pneumonia, surgical site infection, gastrointestinal problems, and mood disorders, usually for reasons such as inappropriate adherence to a therapeutic regimen (generally including medication, dietary, and movement regimens) [2,3]. A lack of, or improper, training of patients may be accompanied by serious complications and re-admissions [3,4], although some of these side effects are preventable. Self-care and understanding of one’s illness, lifestyle changes, and improvement of the patient’s quality of life require the transfer of knowledge and education from the health professional to the patient. Despite recognizing the importance of patient education, several studies have shown that training of these patients may not be effective. Accordingly, effective educational methods should be used for people of different ages and with different levels of literacy to improve the patient’s understanding of the disease condition and the treatment process [5,6].

There are several patient-training methods, such as direct (lectures, individual discussion, group discussion, and teach back) and indirect (booklet, pamphlet, CD, animation-based training, and gamification) methods. The choice of the right educational approach is different depending on the level of literacy and educability of the patient, the mastery of the nurse of the relevant method, and the availability of educational facilities, time, and educational space [7-12]. A number of studies have been conducted on the use of direct methods, such as the teach-back method of patient education [13,14]. This is a comprehensive and evidence-based approach that takes into account health literacy and leads to a better understanding of patients and their caregivers from the training provided by giving them information and asking for them to reflect the key points [15,16]. Despite the demonstrated benefits of this method, studies have shown that nurses are either not using this training method or have not found it to be effective [13,17]. Some of the disadvantages of this method are the time-consuming nature of the training, the large amount of content provided, the lack of repetition of the content at different times, and the lack of skill of the trainer [13,18,19]. In addition, some believe that the increasing speed of science and technology has diminished the role of direct teaching methods in the educational process, emphasizing the use of indirect methods, such as animation-, web-, and smartphone-based training.

In recent years, a new method called gamification has been used as a form of indirect patient training. “Gamification” is** **the term used to define the concept of applying game design and mechanics to nongaming applications. It gives patients the ability to set goals, track progress for achieving them, and get rewarded in return. Gamification also helps to increase users’ self-control and is designed to promote positive behavior change. Gamification has been used to train patients with heart failure, myocardial infarction, rheumatoid arthritis, diabetes, breast cancer, and Alzheimer’s disease and has also been used for smoking cessation and blood pressure control, yielding positive results [20-22]. However, the method has its critics. Some commentators believe that this training method may lead to addictive behavior, that it is costly, that there is a lack of skilled practitioners, and that there is a lack of appropriate infrastructure for its use [22-24].

### Research Aim

Due to the importance of patient education in the development of self-care and the increasing use of modern educational approaches, the aim of this study was to compare the effect of gamification and teach-back training methods on adherence to the therapeutic regimen in patients after CABGS. The primary outcome was a score on adherence to the therapeutic regimen.

## Methods

### Study Design

This randomized clinical trial was performed with a sample size of 123 people in 2019. The study population consisted of all CABGS patients admitted to the intensive care units of hospitals affiliated to the Tehran University of Medical Sciences, Tehran, Iran. The patients who met the study inclusion criteria were randomly divided into three groups of 41 each using randomized block design and software. To decrease the predictability of allocated groups and ensure randomizing participants in an equal number, we used block randomization with size 4. Allocation in each group was random but equal in size.

### Inclusion and Exclusion Criteria

Inclusion criteria were an age range of 18-60 years, an Android phone for the patient, nonuse of psychotropic drugs, the ability to understand and speak Persian, willingness to participate in the study, lack of hearing and speech disorders, and the ability to receive phone calls after discharge. Exclusion criteria were the patient’s unwillingness to continue an education process and acute illness requiring emergency intervention

### Intervention

**Figure 1:**
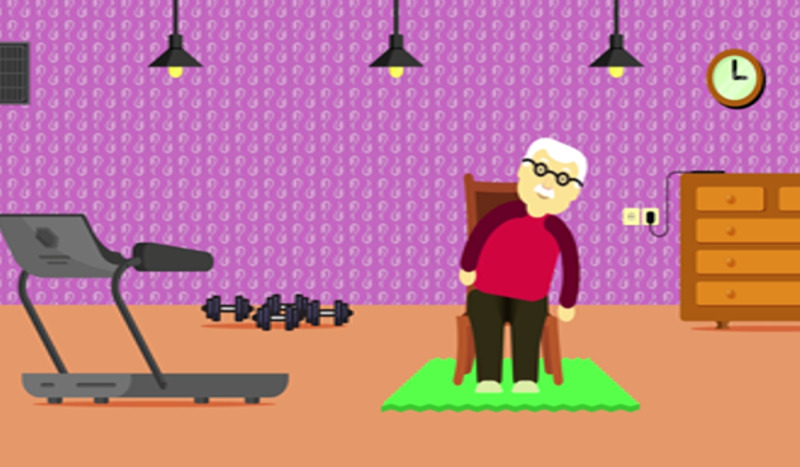
Sample screenshot from the physical activity section.

For patients in the gamification group, informed consent was obtained and a pretest questionnaire completed. The training program was installed on each patient’s smartphone during discharge, and the researcher explained to the participants how to use the game. The app developed for the program included three main sections (dietary regimen, medication regimen, and movement regimen) and one assessment section at the end of each main section. The training was in the form of animation, images, and sound (Figure 1). In the assessment section, which was in the form of a question, the correct answer received 6 stars (reward, motivation); the screen of the phone was full of stars (excitement), and it moved to the next step. If a false answer was given, 3 stars were deducted from the total score (punishment), and the same step was continued until a correct answer was obtained. For a false answer, a sound (eg, a ding) was made (Textbox 1). Each patient was able to see the sum of their scores and those of other patients on the home screen. The patient was also able to see their score chart relative to those of the other participants, and consistent with social comparison theory, thereby motivating them to learn more in comparison to others. The questionnaire was recompleted 30 days later by an in-person visit to the patients’ home.

**Delban (the app, which means “heart protector”) application specifications.**

**Section 1**

Includes the dietary regimen. The scientific content in this section is divided into 7 groups (general recommendations, bread and cereals, meat and beans, dairy, fruits, vegetables, and fat), and finally an assessment is included.

**Section 2**

Includes the medication regimen. General recommendations are given in three subsections for better patient access. The most commonly prescribed heart medications for these patients include β-blockers (eg, Metoral), antihypertensive drugs (eg, Losartan and Valsartan), antiplatelets (eg, Plavix and aspirin), lipid-lowering drugs (eg, Atorvastatin), diuretics (eg, Lasix, Aldactone, triamterene-H, and hydrochlorothiazide), anticoagulants (eg, warfarin and enoxaparin), and vasodilator drugs (eg, SUSTAC). Finally, an assessment is included.

**Section 3**

Includes physical activity. General recommendations are given in three parts. Other areas include walking, using a spirometer, breathing activity, sexual activity, returning to work, driving, and foot edema. In addition, the recommended exercises during the second phase of cardiac rehabilitation after surgery are provided to the patient for 4 weeks. The last part includes an assessment.

**Section 4**

Includes scoring and assessment. The patient’s assessment section answers the questions considered. The correct answer receives 6 stars (reward, motivation), and the screen of the phone is full of stars (excitement), with progression to the next step. In the case of a false answer, 3 stars are deducted from the total score (punishment), and the same step is continued until the answer is correct. For a false answer, a sound (eg, a ding) is made. The patient sees the sum of their scores and those of other patients on the home screen. The patient also sees their score chart relative to the those of others, which motivates them to learn more in comparison to others.

For patients in the teach-back group, after obtaining informed consent and completing the pretest questionnaire, training was presented in the following steps by the researcher: (1) training information in a simple and understandable language without medical terminology, (2) expressing information in the language of the patient without embarrassment, (3) correcting the patient’s misunderstanding, (4) re-asking the patient to make sure the patient was aware of any error, and (5) checking the patient’s correct understanding. The duration of training varied from 45 min to a maximum of 60 min, depending on the patient’s physical and mental condition. In addition, training was provided individually and outside of the inpatient ward, in a separate room, to prevent data transfer to other patients. In addition, a training booklet was given to patients in the teach-back group in order to access information within a 30-day period. Finally, 30 days later, the questionnaire was recompleted by visiting the patient at home, in person. It should be noted that the educational content of both groups was identical.

**Figure 2:**
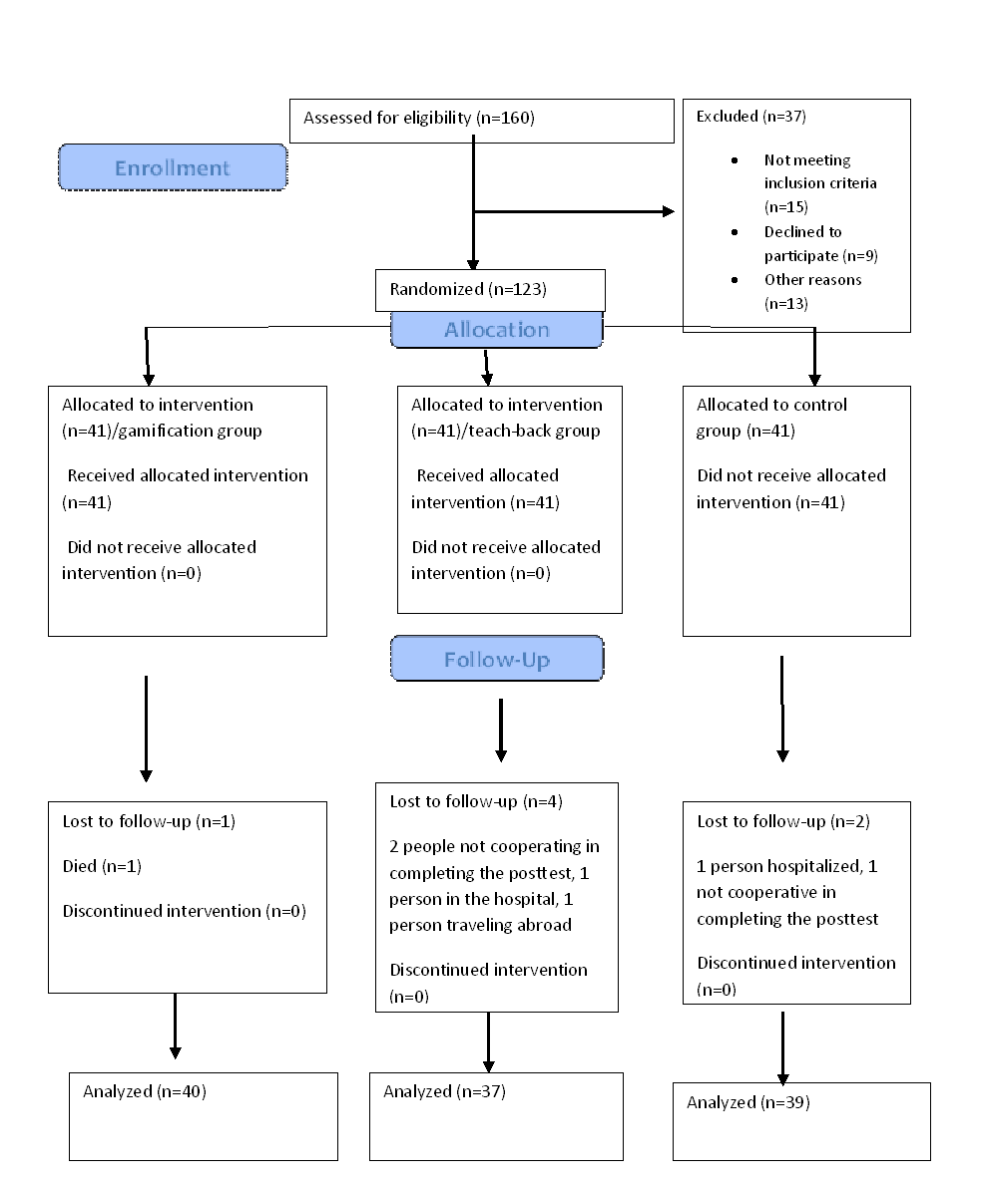
Consolidated Standards of Reporting Trials (CONSORT) flow diagram of this study.

In the control group, no training was provided by the researcher, and the group received only the usual training given by the ward nurse. The questionnaire was completed by the patients before discharge and then 30 days later during a home visit (Figure 2).

### Data Collection Tools

Prior to training, each patient completed an informed consent form, a demographic questionnaire, a questionnaire on adherence to a therapeutic regimen (physical activity and dietary regimen) designed by Sanaie et al [25], and an adherence scale.

The demographic questionnaire included information about age, sex, education, and source of information about the study. In addition, the patient’s health record was referred to obtain details on medical history, past surgery, and type of medication. The questionnaire on adherence to a therapeutic regimen designed by Sanaie et al [25] consists of two sections. The first part contains 30 questions about the patient’s dietary regimen (consumption of salt, fat, meat, dairy, etc). Options (4-point Likert scale) are divided into the number of uses per week, with a total score of 100 points per question. Finally, the total adherence rate of the dietary regimen, where 100% represents a total score of 3000, is decided as follows: <50% of the total score (<1500), undesired adherence; 50%-75% of the total score (1500-2250), relatively desired adherence; and >75% of the total score (>2250), desired adherence.

The second part includes 19 questions about the patient’s movement regimen (eg, walking, breathing exercises, and spirometer). The options are scored from never to always (0 to 110) using a 5-point Likert scale. The total adherence rate of the physical activity regimen, where 100% represents a total score of 1900, is considered as follows: <50% of the total score (<950), undesired adherence; 50%-75% of the total score (950-1425), relatively desired adherence; and >75% of the total score (>1425), desired adherence. The reliability of the questionnaire was measured by the Cronbach alpha value (α=.81).

To confirm content validity, the questionnaire was presented to 10 faculty members of the School of Nursing and Midwifery, Tehran University of Medical Sciences, in addition to a nutritionist, a physiotherapist, a sports medicine specialist, a cardiologist, and an interventional cardiologist. After collecting their comments, corrective and suggested comments were applied.

The MMAS has 7 yes/no options (yes=0 and no=1) and 1 5-point Likert scale (never=0, rarely =1, sometimes=2, often =3, almost always=4). A score of 6 and higher is considered to represent the desired level of adherence to the therapeutic regimen. The MMAS has been translated into Persian by the corresponding author and coauthors, and its validity and reliability (Cronbach α=.82) confirmed [26].

### Statistical Analysis

Data were analyzed with SPSS Statistics version 20 (IBM) and STATA version 12 (StataCorp) using the Fisher exact test, chi-square test, independent t-test, analysis of variance (ANOVA), and Dunnett test.

### Ethical Considerations

This study was approved by the ethics committee of the Tehran University of Medical Sciences, with the code of ethics IR.TUMS.FNM.REC.1398.029, and registered on the database of the Iranian Registry of Clinical Trials (IRCT), with the code IRCT20111203008286N8. Prior to the intervention, the patients signed written informed consent. At the end of the study, the educational content was provided to the control group.

## Results

The results of this study showed that 64.68% of the samples in the gamification group, 51.35% in the teach-back group, and 62.66% in the control group were male. Other demographic characteristics of the patients are listed in Table 1.

In addition, the results of this study showed that the mean (SD) scores of dietary and movement regimen adherence were higher in the gamification group than in the other two groups (Table 2).

**Table 1:** Patients’ baseline characteristics.

Demographic characteristics		Control, n (%)	Gamification, n (%)	Teach back, n (%)	Results		
					χ^2^	*df*	*P* value
**Gender**					1.56	2	.45^a^
	Female	14 (37.8)	13 (35.1)	18 (48.6)			
	Male	23 (62.1)	24 (64.8)	19 (51.3)			
**Educational level**						8	.17^b^
	Primary education	2 (5.4)	5 (13.5)	3 (8.1)			
	High school	4 (10.8)	4 (10.8)	2 (5.4)			
	Diploma	12 (32.4)	10 (27.03)	5 (13.5)			
	Graduate	14 (37.8)	9 (24.3)	21 (56.7)			
	Postgraduate	5 (13.5)	9 (24.3)	6 (16.22)			
**Income status**						4	.88^b^
	It is not enough	28 (75.6)	30 (81)	29 (73.3)			
	It is enough to some extent	6 (16.2)	5 (13.5)	7 (18.9)			
	It is enough	3 (8.1)	2 (5.4)	1 (2.7)			
**Chronic condition**						3	.80^b^
	Diabetes	8 (21.6)	9 (24.3)	7 (18.9)			
	Hyperlipidemia	7 (18.9)	6 (16.2)	10 (27)			
	Hypertension	21 (56/7)	18 (48.6)	16 (43.2)			
	COPD^c^	1 (1.7)	3 (8.1)	2 (5.40)			
	Chronic kidney disease	0 (0)	1 (1.7)	2 (5.40)			
**Source of information**						3	.71^b^
	Physician	8 (21.6)	4 (10.8)	2 (5.4)			
	Nurse	8 (21.6)	3 (8.1)	1 (2.7)			
	Media	3 (8.1)	3 (8.1)	8 (21.6)			
	Social network and the internet	18 (48.6)	28 (72.9)	26 (70.2)			
**Fat consumption**					374/4	2	.12^a^
	Low fat	13 (35.1)	7 (18.9)	6 (16.2)			
	Ordinary fat	14 (37.8)	27 (67.5)	17 (45.9)			
	Fatty	10 (27)	5 (13.5)	14 (37.8)			
**Salt consumption**					240/0	2	.88^a^
	Low salt	13 (35.1)	7 (18.9)	6 (16.21)			
	Ordinary salt	14 (37.8)	25 (67.5)	17 (45.9)			
	Salty	10 (27.0)	5 (13.5)	14 (37.8)			

^a^Chi-squared test.

^b^The Fisher exact text.

^c^COPD: chronic obstructive pulmonary disease.

**Table 2:** Comparison of mean (SD) scores of dietary and movement regimen adherence in the study groups.

Study group		Mean (SD)	95% CI^a^
**Diet regimen**			
	Control	–0.7639 (0.5200)	(–0.9885, –0.5393)
	Gamification	1.033 (0.655)	(0.815, 1.251)
	Teaching	–0.347(0.700)	(–0.575, –0.119)
**Movement regimen (physical activity)**			
	Control	–0.9095 (0.3253)	(–1.0961, –0.7229)
	Gamification	1.104 (0.653)	(0.923, 1.285)
	Teaching	–0.2745 (0.5270)	(–0.4641, –0.0848)
**Medication regimen**			
	Control	–0.398 (0.679)	(–0.726, –0.071)
	Gamification	0.555 (0.924)	(0.238, 0.873)
	Teaching	–0.200 (1.107)	(–0.533, 0.133)

^a^CI: confidence interval.

**Figure 3:**
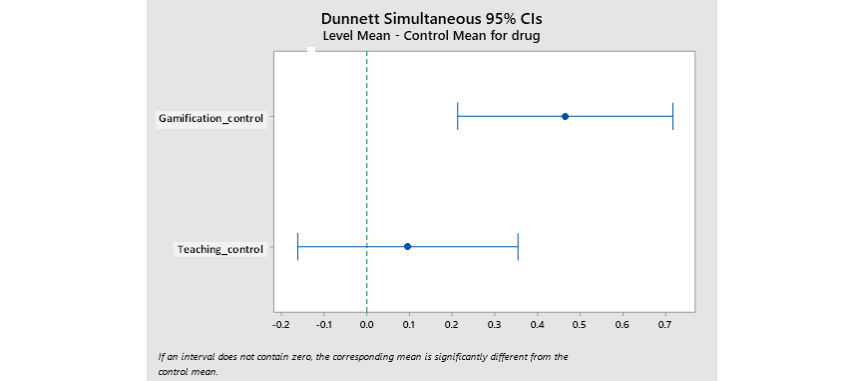
Comparison of CIs between intervention groups (gamification and teach back) and the control group. CI: confidence interval.

Initially, for each outcome, the analysis was performed without dropout management and then with dropout data considered. The results of the analysis showed that no significant difference was found. The implication of these results is that dropout did not play a role in the significance level of the study results. Moreover, the one-way ANOVA test for comparing the mean scores of teach-back and gamification training methods showed that the mean normalized scores for the dietary regimen (*P*<.001, *F*=71.80), movement regimen (*P*<.001, *F*=124.53), and medication regimen (*P*<.001, *F*=9.66) before and after the intervention were significantly different between teach-back, gamification, and control groups. After the heterogeneity of these scores was determined, the Dunnett test was used to compare teach-back and gamification groups with the control group. In addition, in the dietary and movement regimen adherence, there was no overlap in the gamification method with the teach-back method due to the confidence intervals (CIs) of the mean difference with the control group, which indicates that the gamification approach performs significantly better than the teach-back method (Table 3). In the adherence to the therapeutic regimen, the CIs of the mean difference with the control group overlapped in the gamification approach (0.438, 1.469) and the teach-back approach (-0.330, 0.727), which shows that the gamification approach is not significantly different from the teach-back approach (Figure 3).

**Table 3:** Dunnett simultaneous tests for level mean–control mean.

Difference of levels		Mean difference^a^	SE^b^ of difference	95% CI^c^		*P* value	
**Diet regimen**							
	Gamification-control	1.797	0.157		(1.443, 2.151)		<.001
	Teaching-control	0.417	0.161		(0.055, 0.779)		.021
**Movement regimen (physical activity)**							
	Gamification-control	2.013	0.131		(1.719, 2.307)		<.001
	Teaching-control	0.635	0.134		(0.334, 0.936)		<.001
**Medication regimen**							
	Gamification-control	0.954	0.230		(0.438, 1.469)		<.001
	Teaching-control	0.199	0.235		(-0.330, 0.727)		.61

^a^Individual confidence level=97.29%.

^b^SE: standard error.

^c^CI: confidence interval.

## Discussion

### Principal Findings

This clinical trial was conducted in 2019 with the aim of determining and comparing the effects of gamification and teach-back training methods on adherence to a therapeutic regimen by patients after CABGS. The study found that the gamification training method performs better than the teach-back training method in dietary and movement regimen adherence, but there was no statistically significant difference in adherence to a medication regimen between the two groups. In addition, sensitivity analysis results showed that the dropout did not play a significant role in the significance level of the study results.

A study by Ghanbari et al [27] on the effect of an educational program based on the teach-back method on adherence to a treatment regimen in patients with end-stage renal disease on dialysis showed that the mean score of treatment adherence in 3 areas of hemodialysis, namely drug therapy, fluid restriction, and dietary regimen, in the intervention group in both the posttest (7 days after intervention) and follow-up (30 days after intervention) was significantly higher than in the control group (*P*<0.01) [27]. In this study, the use of a teach-back training method in relation to movement and dietary regimens showed adherence to the therapeutic regimen, a finding that is in line with Ghanbari et al [27]. However, the use of the teach-back training method in relation to adherence to the medication regimen is inconsistent with their findings. The inconsistency between the results of Ghanbari et al [27] and this study may be due to a longer period of treatment and the periodic nature of hemodialysis compared with open heart surgery and the repetition of teaching during hemodialysis treatment, thus producing a more positive result. In addition, since the medication regimen is such an important pillar of treatment adherence in patients on hemodialysis, more attention may be paid to this area of treatment in patient education using additional information resources, including other patients and treatment staff. Dalir et al’s [28] study of the effect of a teach-back intervention aimed at improving self-care in 62 patients with heart failure found that the teach-back training method is effective in improving self-care (*P*<0.001) [28]. Our study was able to show improvements in relation to movement and diet; however, it did not have the same positive result on the medication regimen that was demonstrated by Dalir et al [28]. The lack of effectiveness in relation to medication adherence in this study may be attributed to the duration of the training. In the Dalir et al [28] study, the patients were taught over 3-4 days, while in this study, the training was presented in a single 45-60-min session. Other studies have also shown the benefits of longer periods of patient education using teach-back training methods. These include White et. al’s [29] study of patients with heart failure, whose education occurred over a 13-month period when hospitalized.

Finally, in relation to previous research on gamification effects, Allam et al’ [30] RCT on the effect of social support features and gamification on a web-based intervention for patients with rheumatoid arthritis found that physical activity increased in patients in the intervention group who had access to gamification and social support. The use of health care decreased for patients who received social support and for those who received social support and gamification. The study showed that the use of gamification alone or in conjunction with website intervention increases physical activity as well [30]. These significant differences in relation to physical activity using the gamification approach are consistent with our study, as are design elements such as the use of game elements to create excitement and motivation and the awarding of prizes to the “winning” patients.

### Limitations

We had to rely on the patients’ self-report on exercising at home and following the dietary and medication regimens. In addition, the patients did not record the number of games and browse the application. The app did not include the feature to send reminders and the ability for patients to interact with it. We suggest further studies with a larger sample size and an interactive app with the ability to record usage and to send reminders. In addition, it is recommended that studies be performed in the presence of active family member inpatient care using these approaches.

### Conclusion

Using a game-based smartphone app as a support program to educate patients can affect the patients’ adherence to their therapeutic regimen. In addition, with this program, the patients can access the training on their smartphones at any time and place and can repeat the instructions, if necessary. The combination of game elements and patient education together can lead to a better, more engaging learning experience, faster feedback, and more readily available reminders of educational content. However, according to social factor theory, the social signs in multimedia messages (eg, the presentation of an educational agent along with a human voice) causes learners to consider computer-centered learning environments as discourse environments. Signs that speak in the form of a friendly factor on the monitor screen with a human voice and movements increase the ability to transmit positively. The theory of social mediation suggests that bringing verbal (eg, spoken words) and nonverbal (eg, gestures, gaze, and movement) social cues into multimedia environments can simulate human-to-human communication, facilitating engagement of learners in the learning process. According to this theory, by combining a multimedia learning environment and a moving factor as a visual and verbal social symbol, virtual communication between that factor and learners becomes a suitable alternative for human interactions. This study showed that the use of new technology-based approaches can replace previous educational methods. Therefore, by using this method, the process of educating patients can be made more up to date and more attractive by making optimal use of smartphones. Based on the positive results of this study, the teach-back training method is a strategy that can used to increase patients’ understanding and is widely accepted by health care organizations as an effective way to communicate information. Easy access, low cost, and the interactive nature of this method are other notable benefits. Training ends when the patient reaches an acceptable level of understanding of the subject. The teach-back approach can be considered an effective and alternative training method instead of traditional ones, such as pamphlets and booklets.

## Appendix

Multimedia Appendix 1 CONSORT-eHEALTH checklist (V 1.6.1).

## Abbreviations

ANOVA: analysis of variance
CABGS: coronary artery bypass graft surgery
CI: confidence interval
COPD: chronic obstructive pulmonary disease
