# Supplementary material for: Comparing the Effects of Gamification and Teach-Back Training Methods on Adherence to a Therapeutic Regimen in Patients After Coronary Artery Bypass Graft Surgery: Randomized Clinical Trial
Source: J Med Internet Res. 2021 Dec 10;23(12):e22557. doi: 10.2196/22557 (PMC8709912; doi:10.2196/22557)
Supplement: Multimedia Appendix 1 [file jmir_v23i12e22557_app1.pdf]

CONSORT-EHEALTH Checklist V1.6.2 Report

Manuscript Number 22557

(based on CONSORT-EHEALTH V1.6), available at [http://tinyurl.com/consort-ehealth-v1-6].

Date completed
11/14/2021 11:55:43
by
Fatemeh Bahramnezhad

Comparing the Effects of Gamification and Teach-Back Training Methods on Adherence to a Therapeutic Regimen in Patients After Coronary Artery Bypass Graft Surgery: Randomized Clinical Trial

TITLE

1a-i) Identify the mode of delivery in the title
we use gamification in title
1a-ii) Non-web-based components or important co-interventions in title

1a-iii) Primary condition or target group in the title
in Patients
After Coronary Artery Bypass Graft Surgery

ABSTRACT

1b-i) Key features/functionalities/components of the intervention and comparator in the METHODS section of the ABSTRACT
Effect of gamification and teach-back training methods
1b-ii) Level of human involvement in the METHODS section of the ABSTRACT

1b-iii) Open vs. closed, web-based (self-assessment) vs. face-to-face assessments in the METHODS section of the ABSTRACT

1b-iv) RESULTS section in abstract must contain use data

1b-v) CONCLUSIONS/DISCUSSION in abstract for negative trials

INTRODUCTION

2a-i) Problem and the type of system/solution
Compare the effect of gamification and teach-back training methods on adherence to a therapeutic regimen in patients after CABGS
2a-ii) Scientific background, rationale: What is known about the (type of) system
Due to the importance of patient education in the development of self-care and the increasing use of modern educational approaches, the aim of this study was to compare the effect of gamification and teach-back training methods on adherence to the therapeutic regimen in patients after CABGS. The primary outcome was a score on adherence to the therapeutic regimen.
Does your paper address CONSORT subitem 2b?
Due to the importance of patient education in the development of self-care and the increasing use of modern educational approaches, the aim of this study was to compare the effect of gamification and teach-back training methods on adherence to the therapeutic regimen in patients after CABGS. The primary outcome was a score on adherence to the therapeutic regimen

METHODS

3a) CONSORT: Description of trial design (such as parallel, factorial) including allocation ratio
The patients who met the study inclusion criteria were randomly divided into three groups of 41 each using randomized block design and software. To decrease the predictability of allocated groups and ensure randomizing participants in an equal number, we used block randomization with size 4. Allocation in each group was random but equal in size.
3b) CONSORT: Important changes to methods after trial commencement (such as eligibility criteria), with reasons
Inclusion criteria were an age range of 18-60 years, an Android phone for the patient, nonuse of psychotropic drugs, the ability to understand and speak Persian, willingness to participate in the study, lack of hearing and speech disorders, and the ability to receive phone calls after discharge. Exclusion criteria were the patient's unwillingness to continue an education process and acute illness requiring emergency intervention
3b-i) Bug fixes, Downtimes, Content Changes

4a) CONSORT: Eligibility criteria for participants
an Android phone for the patient,the ability to receive phone calls after discharge
4a-i) Computer / Internet literacy

4a-ii) Open vs. closed, web-based vs. face-to-face assessments:
They acess offline. For patients in the gamification group, informed consent was obtained and a pretest questionnaire completed. The training program was installed on each patient's smartphone during discharge, and the researcher explained to the participants how to use the game.
4a-iii) Information giving during recruitment

4b) CONSORT: Settings and locations where the data were collected
Authors don't mention any conflict of interst
4b-i) Report if outcomes were (self-)assessed through online questionnaires
Prior to training, each patient completed an informed consent form, a demographic questionnaire, a questionnaire on adherence to a therapeutic regimen (physical activity and dietary regimen) designed by Sanaie et al , and Adherence Scale
4b-ii) Report how institutional affiliations are displayed

5) CONSORT: Describe the interventions for each group with sufficient details to allow replication, including how and when they were actually administered
5-i) Mention names, credential, affiliations of the developers, sponsors, and owners

5-ii) Describe the history/development process

5-iii) Revisions and updating

5-iv) Quality assurance methods

5-v) Ensure replicability by publishing the source code, and/or providing screenshots/screen-capture video, and/or providing flowcharts of the algorithms used

5-vi) Digital preservation

5-vii) Access
The reliability of the questionnaires was measured by the Cronbach alpha value (α=.81).

To confirm content validity, the questionnaires was presented to 10 faculty members of the School of Nursing and Midwifery, Tehran University of Medical Sciences, in addition to a nutritionist, a physiotherapist, a sports medicine specialist, a cardiologist, and an interventional cardiologist. After collecting their comments, corrective and suggested comments were applied.

5-viii) Mode of delivery, features/functionalities/components of the intervention and comparator, and the theoretical framework
The questionnaire was completed by the patients n three groups before discharge and then 30 days later during a home visit
5-ix) Describe use parameters

5-x) Clarify the level of human involvement

**5-xi) Report any prompts/reminders used**		
The researchers sent reminder text messages to participants in the teachback and gamification group each week		
**5-xii) Describe any co-interventions (incl. training/support)**		
We didn't have any additional education		
**6a) CONSORT: Completely defined pre-specified primary and secondary outcome measures, including how and when they were assessed**		
The demographic questionnaire included information about age, sex, education, and source of information about the study. In addition, the patient's health record was referred to obtain details on medical history, past surgery, and type of medication. The questionnaire on adherence to a therapeutic regimen designed by Sanaie et al.The second part includes 19 questions about the patient's movement regimen (eg, walking, breathing exercises, and spirometer). The options are scored from never to always (0 to 110) using a 5-point Likert scale. The total adherence rate of the physical activity regimen, where 100% represents a total score of 1900, is considered as follows: <50% of the total score (<950), undesired adherence; 50%-75% of the total score (950-1425), relatively desired adherence; and >75% of the total score (>1425), desired adherence.		
**6a-i) Online questionnaires: describe if they were validated for online use and apply CHERRIES items to describe how the questionnaires were designed/deployed**		
**6a-ii) Describe whether and how "use" (including intensity of use/dosage) was defined/measured/monitored**		
**6a-iii) Describe whether, how, and when qualitative feedback from participants was obtained**		
**6b) CONSORT: Any changes to trial outcomes after the trial commenced, with reasons**		
Authors don't mention any conflict of interst		
**7a) CONSORT: How sample size was determined**		
**7a-i) Describe whether and how expected attrition was taken into account when calculating the sample size**		
**7b) CONSORT: When applicable, explanation of any interim analyses and stopping guidelines**		
The demographic questionnaire included information about age, sex, education, and source of information about the study. In addition, the patient's health record was referred to obtain details on medical history, past surgery, and type of medication. The questionnaire on adherence to a therapeutic regimen designed by Sanaie et al.The second part includes 19 questions about the patient's movement regimen (eg, walking, breathing exercises, and spirometer). The options are scored from never to always (0 to 110) using a 5-point Likert scale. The total adherence rate of the physical activity regimen, where 100% represents a total score of 1900, is considered as follows: <50% of the total score (<950), undesired adherence; 50%-75% of the total score (950-1425), relatively desired adherence; and >75% of the total score (>1425), desired adherence.		
**8a) CONSORT: Method used to generate the random allocation sequence**		
The patients who met the study inclusion criteria were randomly divided into three groups of 41 each using randomized block design and software. To decrease the predictability of allocated groups and ensure randomizing participants in an equal number, we used block randomization with size 4. Allocation in each group was random but equal in size.		
**8b) CONSORT: Type of randomisation; details of any restriction (such as blocking and block size)**		
The patients who met the study inclusion criteria were randomly divided into three groups of 41 each using randomized block design and software. To decrease the predictability of allocated groups and ensure randomizing participants in an equal number, we used block randomization with size 4. Allocation in each group was random but equal in size.		
**9) CONSORT: Mechanism used to implement the random allocation sequence (such as sequentially numbered containers), describing any steps taken to conceal the sequence until interventions were assigned**		
Mechanism used to implement<br>the random allocation sequence<br>(such as sequentially numbered<br>containers), describing any steps<br>taken to conceal the sequence<br>until interventions were assigned		
**10) CONSORT: Who generated the random allocation sequence, who enrolled participants, and who assigned participants to interventions**		
Mechanism used to implement<br>the random allocation sequence<br>(such as sequentially numbered<br>containers), describing any steps<br>taken to conceal the sequence<br>until interventions were assigned		
**11a) CONSORT: Blinding - If done, who was blinded after assignment to interventions (for example, participants, care providers, those assessing outcomes) and how**		
**11a-i) Specify who was blinded, and who wasn't**		
We didn't have blind participants		
**11a-ii) Discuss e.g., whether participants knew which intervention was the "intervention of interest" and which one was the "comparator"**		
**11b) CONSORT: If relevant, description of the similarity of interventions**		
We didn't have this item in this study		
**12a) CONSORT: Statistical methods used to compare groups for primary and secondary outcomes**		
Data were analyzed with SPSS Statistics version 20 (IBM) and STATA version 12 (StataCorp) using the Fisher exact test, chi-square test, independent t-test, analysis of variance (ANOVA), and Dunnett test.		
**12a-i) Imputation techniques to deal with attrition / missing values**		
Data were analyzed with SPSS Statistics version 20 (IBM) and STATA version 12 (StataCorp) using the Fisher exact test, chi-square test, independent t-test, analysis of variance (ANOVA), and Dunnett test. we didn't have missing values		
**12b) CONSORT: Methods for additional analyses, such as subgroup analyses and adjusted analyses**		
We didn't have subgroup nalysis		
RESULTS		
**13a) CONSORT: For each group, the numbers of participants who were randomly assigned, received intended treatment, and were analysed for the primary outcome**		
a sample size of 123 people (41 people in each group(		
**13b) CONSORT: For each group, losses and exclusions after randomisation, together with reasons**		
We mentioned the number and cause of sample loss in each group separately		
**13b-i) Attrition diagram**		
**14a) CONSORT: Dates defining the periods of recruitment and follow-up**		
. In addition, training was provided individually and outside of the inpatient ward, in a separate room, to prevent data transfer to other patients. In addition, a training booklet was given to patients in the teach-back group in order to access information within a 30-day period. Finally, 30 days later, the questionnaire was recompleted by visiting the patient at home, in person. It should be noted that the educational content of both groups was identical.		
**14a-i) Indicate if critical "secular events" fell into the study period**		
**14b) CONSORT: Why the trial ended or was stopped (early)**		
We reached the desired sample size and sampling stopped		
**15) CONSORT: A table showing baseline demographic and clinical characteristics for each group**		
The results of this study showed that 64.68% of the samples in the gamification group, 51.35% in the teach-back group, and 62.66% in the control group were male. Other demographic characteristics of the patients are listed in Table 1.		
**15-i) Report demographics associated with digital divide issues**		
Age, Gender, Educational Statuse, Income status,Chronic condition,Source of information ,...reported		
**16a) CONSORT: For each group, number of participants (denominator) included in each analysis and whether the analysis was by original assigned groups**		
**16-i) Report multiple "denominators" and provide definitions**		
We reported P-value, Confidence interval and Mean(sd)		
**16-ii) Primary analysis should be intent-to-treat**		
**17a) CONSORT: For each primary and secondary outcome, results for each group, and the estimated effect size and its precision (such as 95% confidence interval)**		

We reported for each primary and secondary<br>outcome, results for each group,<br>and the estimated effect size and<br>its precision (such as 95%<br>confidence interval)		
**17a-i) Presentation of process outcomes such as metrics of use and intensity of use**		
**17b) CONSORT: For binary outcomes, presentation of both absolute and relative effect sizes is recommended**		
Not applicable in the present study		
**18) CONSORT: Results of any other analyses performed, including subgroup analyses and adjusted analyses, distinguishing pre-specified from exploratory**		
We didn't have subgroup analysis		
**18-i) Subgroup analysis of comparing only users**		
**19) CONSORT: All important harms or unintended effects in each group**		
We had no harms in this study		
**19-i) Include privacy breaches, technical problems**		
**19-ii) Include qualitative feedback from participants or observations from staff/researchers**		
DISCUSSION		
**20) CONSORT: Trial limitations, addressing sources of potential bias, imprecision, multiplicity of analyses**		
**20-i) Typical limitations in ehealth trials**		
We had to rely on the patients' self-report on exercising at home and following the dietary and medication regimens. In addition, the patients did not record the number of games and browse the application. The app did not include the feature to send reminders and the ability for patients to interact with it. We suggest further studies with a larger sample size and an interactive app with the ability to record usage and to send reminders. In addition, it is recommended that studies be performed in the presence of active family member inpatient care using these approaches.		
**21) CONSORT: Generalisability (external validity, applicability) of the trial findings**		
**21-i) Generalizability to other populations**		
**21-ii) Discuss if there were elements in the RCT that would be different in a routine application setting**		
**22) CONSORT: Interpretation consistent with results, balancing benefits and harms, and considering other relevant evidence**		
**22-i) Restate study questions and summarize the answers suggested by the data, starting with primary outcomes and process outcomes (use)**		
In the discussion, the results of the present study were compared with the results of other studies		
**22-ii) Highlight unanswered new questions, suggest future research**		
Other information		
**23) CONSORT: Registration number and name of trial registry**		
This study registered on the database of the Iranian Registry of Clinical Trials (IRCT), with the code IRCT20111203008286N8		
**24) CONSORT: Where the full trial protocol can be accessed, if available**		
Yes,full trial protocol can<br>be accessed, if available		
**25) CONSORT: Sources of funding and other support (such as supply of drugs), role of funders**		
Tehran University of Medical Sciences was funder		
**X26-i) Comment on ethics committee approval**		
**x26-ii) Outline informed consent procedures**		
**X26-iii) Safety and security procedures**		
**X27-i) State the relation of the study team towards the system being evaluated**		
